# Herpesvirus Infections After Chimeric Antigen Receptor T-Cell Therapy and Bispecific Antibodies: A Review

**DOI:** 10.3390/v17010133

**Published:** 2025-01-18

**Authors:** Joseph Sassine, Emily A. Siegrist, Roy F. Chemaly

**Affiliations:** 1 Infectious Diseases Section, Department of Medicine, University of Oklahoma Health Sciences Center, Oklahoma City, OK 73104, USA; 2 Department of Pharmacy, OU. Health, Oklahoma City, OK 73104, USA; 3 Department of Infectious Diseases, Infection Control, and Employee Health, The University of Texas MD Anderson Cancer Center, 1515 Holcombe Boulevard, Houston, TX 77030, USA

**Keywords:** herpesviruses, CAR T cell therapy, bispecific antibodies, HSV, VZV, CMV, HHV-6

## Abstract

In this narrative review, we explore the burden and risk factors of various herpesvirus infections in patients receiving chimeric antigen receptor T-cell (CAR-T) therapy or bispecific antibodies (BsAb) for the treatment of hematologic malignancies. Antiviral prophylaxis for herpes simplex/varicella zoster viruses became part of the standard of care in this patient population. Breakthrough infections may rarely occur, and the optimal duration of prophylaxis as well as the timing of recombinant zoster immunization remain to be explored. Clinically significant cytomegalovirus (CMV) infections can affect up to 10% of patients after CAR-T, depending on the CAR-T product target, post-CAR-T complications such as cytokine release syndrome and the need for glucocorticoid therapy. Surveillance and prophylactic strategies for CMV need to be developed, whereas the risk factors for and the burden of CMV infections after BsAb are not yet well-defined. Human herpes virus 6 reactivation and end organ disease such as encephalitis are rarely reported after CAR-T and have not yet been reported after BsAb; additional research is needed.

## 1. Introduction

Herpesvirus infections represent a significant infectious complication after hematopoietic cell transplantation (HCT) and can significantly affect patients with hematologic malignancies [1]. As new therapeutic modalities for hematologic malignancies emerge, such as chimeric antigen receptor T-cell (CAR-T) therapy or bispecific antibodies (BsAb), our understanding of the burden and risk factors for herpesvirus reactivations in patients receiving these novel therapies remains limited. This narrative review explores the available data on the incidence and risk factors for herpes simplex virus (HSV), varicella zoster virus (VZV), cytomegalovirus (CMV) and human herpesvirus 6 (HHV-6) in patients receiving currently approved CAR-T therapy or BsAb for the treatment of hematologic malignancies, reviewing current recommendations on prevention, management and monitoring and offering directions for future research.

## 2. Herpesviruses and Chimeric Antigen Receptor T-Cell (CAR-T) Therapy

Herpesvirus reactivation has been reported since the early clinical trials of CD19 CAR-T in patients with B-ALL and B-cell lymphomas. One of the first studies of infectious complications of CD19 CAR-T by Hill et al. evaluated 133 patients enrolled in a phase 1/2 study of CD19 CAR-T, all of whom received acyclovir or valacyclovir for HSV/VZV prophylaxis, and one patient had CMV reactivation without end organ disease in the first 28 days after CAR-T [2]. Between day 29 and 90 post CAR-T, one patient developed CMV reactivation, and another one developed CMV pneumonitis, neither of whom had a history of HCT [2]. Additional retrospective studies seemed to reflect a low incidence of herpesvirus reactivation after CD19 CAR-T, with occasional cases of HSV/VZV, most of whom were not on acyclovir prophylaxis, and a few cases of CMV DNAemia [3,4,5]. Nevertheless, a study of 41 patients with large B-cell lymphoma suggested a more significant burden of herpesviruses, affecting a quarter of their cohort, with one case of HHV-6 meningoencephalitis in the first 28 days, four cases of CMV reactivation within two weeks of glucocorticoid initiation, and six cases of herpes zoster occurring beyond 28 days post CD19 CAR-T [6].

The above findings led to the recognition of herpesvirus reactivation as a potential infectious complication of CD19 CAR-T and, by extension, of B-Cell Maturation Antigen (BCMA) CAR-T, where the data are more limited. In addition to the on-target, off-tumor effects of both CD19 and BCMA CAR-T causing B-cell/plasma cell aplasia and subsequent hypogammaglobulinemia [7], significant impairment in T-cell-mediated immunity occurs. Administration of lymphodepletion chemotherapy, typically with fludarabine and cyclophosphamide, precedes the infusion of CAR-T [8]. The nature of the lymphodepleting chemotherapy regimen, particularly the dose of cyclophosphamide, affects infection risk [3]. Additionally, a significant proportion of patients develop cytokine release syndrome (CRS) and immune-effector cell-associated neurotoxicity syndrome (ICANS) after CAR-T, the treatment of which requires further immunosuppression, with tocilizumab, anakinra and glucocorticoids [8]. Furthermore, many patients, particularly early after CAR-T approval, received the latter after failing multiple lines of therapy, including autologous and allogeneic HCT (36–55% of patients in some of the aforementioned cohorts [2,3,4]); thus, these patients entered the CAR-T stage of their treatment with an already unfavorable net state of immunosuppression. While this might not be the case for some CD19 CAR-T recipients as those products are used earlier as lines of treatment, this remains an important factor for recipients of BCMA CAR-T. These factors may lead to a slow recovery of T-cell-mediated immunity after CAR-T, as demonstrated by prolonged CD4 lymphopenia [9], an established pathophysiological mechanism and immunological indicator of risk for herpesvirus reactivations after allogeneic HCT [1]. A study of 31 patients who received CD19 CAR-T for B-cell lymphoma under clinical trial showed delayed CD4 recovery in three out of nine patients tested one year after CAR-T, and in two out of seven patients tested two years after CAR-T [9]. A large retrospective study evaluated 160 patients who received FDA-approved CD19 CAR-T and identified grade 3/4 CRS, grade 3/4 ICANS, a higher cumulative dose of glucocorticoids in the first 30 days, and administration of anakinra as independent risk factors for herpesvirus reactivation [10]. Additionally, a large database analysis, including 2256 patients who received CD19 or BCMA CAR-T, reported a 13.6% prevalence of herpesvirus reactivations, occurring at a median of 71 days post CAR-T (IQR 18–252 days), with CMV being the most common (7.5% of patients) followed by other herpesviruses (<3% of patients each) [11]. Independent risk factors for herpesvirus reactivations in this study included prior HCT, HIV, hypogammaglobulinemia, ICANS, hemophagocytic lymphohistiocytosis, rituximab and anakinra [11]. Patients with diffuse large B-cell lymphoma or mantle cell lymphoma were associated with a lower risk of herpesvirus reactivations [11].

In this section, we will review specific studies regarding HSV/VZV, CMV and HHV-6 after CAR-T. We excluded EBV as the clinical significance of EBV detection after CAR-T in patients with B-cell malignancies remains to be determined. It is worthwhile noting that CD19 CAR-T products have been used in the treatment of post-transplant lymphoproliferative disease (PTLD) [12], but no cases of PTLD occurring after autologous CAR-T have been reported so far.

### 2.1. HSV/VZV

The risk of HSV and VZV reactivations after CAR-T is widely acknowledged, and many institutions implemented antiviral prophylaxis as standard of care, which is reflected in the high rates of antiviral prophylaxis in published clinical trials and real-world data [2,4,5,10]. The majority of reported cases of HSV and VZV reactivations after CAR-T occurred in patients who were not on antiviral prophylaxis in the earliest clinical trials [3,4,9]. In a cohort of 31 patients who received CD19 CAR-T under a clinical trial without antiviral prophylaxis, five events of VZV reactivation and three events of HSV reactivation were reported [9]. Hence, most institutions and professional societies recommend antiviral prophylaxis, including the American Society of Transplant and Cellular Therapy (ASTCT) which recommends acyclovir or valacyclovir from the initiation of lymphodepletion until at least 6 months post CAR-T [13]. Although prophylaxis is common, breakthrough infections have been rarely reported [5], including severe complications such as VZV retinitis [14] and HSV pneumonia [15]. As our understanding of the immune reconstitution after CAR-T evolves, the optimal duration of antiviral prophylaxis remains to be fully determined and the role of CD4 count monitoring as a surrogate marker of immune reconstitution may warrant additional studies.

There are limited data on the serologic response to the recombinant zoster vaccine after CAR-T. While a diminished antibody response is anticipated after both CD19 and BCMA CAR-T due to on-target, off-tumor effects on B lymphocytes and plasma cells, the pathogen-specific antibody response is more significantly impaired after BCMA CAR-T compared to CD19 CAR-T, likely due to the impact of BCMA CAR-T on antibody-producing plasma cells [7]. The pathogen-specific antibody response is likely preserved after CD19 CAR-T, especially in the absence of prior allogeneic HCT [16]. A prospective, cross-sectional study that evaluated vaccine-preventable diseases including VZV showed that CD19 CAR-T recipients had IgG levels correlating with seroprotection comparable to the general population, whereas BCMA CAR-T recipients were about 50% less likely to achieve these IgG levels, with fewer pathogen-specific epitope hits compared to CD19 CAR-T recipients [17]. The ASTCT recommends the recombinant zoster vaccine (Shingrix^®^) for VZV-seropositive adult CAR-T recipients or those with prior varicella or zoster infections [13], although the efficacy and the right schedules still need to be determined. The proposed schedule based on the guidelines suggested that the recommended first and second doses be administered at least 12 and 18 months post-CAR-T, provided the patient is >1 year post HCT, >8 months off systemic immunosuppressive therapy and with an absolute CD4 count > 200 cells/µL [13]. This immunization schedule in relation with the timing of antiviral prophylaxis discontinuation needs further investigations.

### 2.2. CMV

CAR-T recipients vary in terms of baseline risk for CMV prior to infusion. While those who receive CAR-T for B-ALL are more likely to have had prior allogeneic HCT and thus be at higher risk for CMV [1], the latter has not been reported as a common or frequent infectious complication of lymphoma or multiple myeloma therapy prior to the CAR-T era. In a prior study by our group at a large comprehensive cancer center over a 4-year period, only 84 patients with lymphoma or multiple myeloma on different lines of therapy developed clinically significant CMV infection (CS-CMVi is defined as CMV end organ disease and/or CMV DNAemia leading to preemptive antiviral therapy based on prespecified thresholds [18,19]. Nevertheless, CS-CMVi carried). Nevertheless, CS-CMVi carried significant morbidity and mortality in this cohort, as 63% of the patients were diagnosed with CMV end organ disease, 19% had recurrent CS-CMVi and 7% had CMV-attributable mortality [20]. The high proportion of CMV end organ disease could be partly explained by the lack of prospective monitoring for CMV in the plasma or blood, with subsequent delay in antiviral therapy that could have prevented progression to pneumonia, in particular. Within this context, earlier studies in CAR-T recipients reported low rates of CMV reactivation with almost no CMV end organ disease [2,3,4,5], although few case reports described CMV end organ disease such as pneumonia [15,21] and retinitis in this patient population [22,23]. These clinical observations prompted further studies of CMV after CAR-T, most of which are retrospective and are limited by a heterogeneity in CMV surveillance protocols and the CMV viral load thresholds to initiate preemptive antiviral therapy [24,25,26,27,28,29] (Table 1).

One of the largest retrospective cohort studies examined CMV infections in 230 CD19 CAR-T recipients over a 3-year period, with weekly plasma CMV PCR performed in patients with neutropenia or those with grade 3/4 CRS/ICANS for up to one month after CAR-T [24]. CMV reactivation at any level occurred in 51 (22%) patients; 22 (10%) had CS-CMVi at a median of 17 days (range 0–343 days) after CAR-T [24]. Interestingly, 7 out of these 22 (33%) patients developed CMV end organ disease [24]. Independent risk factors for CS-CMVi included Asian or Middle Eastern ethnicity (HR 13.71), treatment of CRS or ICANS with steroids (HR 6.25) and being monitored for CMV reactivation by PCR (HR 6.91) [24]. Similar findings in terms of prevalence and burden of CMV infections after CAR-T were reported in other studies [11,25,26,27,28,29] and are summarized in Table 1. Overall, CMV reactivations happen early, at a median of 14–21 days [25,27,28,29], in up to half of the patients; however, CS-CMVi was significantly less common, with prevalence ranging between 3% and 15% (Table 1). Risk factors for any CMV reactivation included two or more immunosuppressants [25], dexamethasone [27] or the use of axicabtagene cileucel (attributed to a higher rate of CRS with this product) [28]. In a recent large database analysis of viral infections after CAR-T, the prevalence of CMV reactivation was 7.5%, occurring at a median of 60 days (IQR 20–215), earlier than all other viral infections reported in this study, and with a median CMV viral load of 1719 IU/mL (IQR 808–7500 IU/mL) [11]. In a prospective study of 72 CMV-seropositive adults who received CD19, CD20 or BCMA-CAR-T and had surveillance with plasma CMV PCR at baseline and weekly up to 12 weeks post CAR-T, the cumulative incidence of CMV reactivation was 27% by week 12, with a median CMV viral load of 127 IU/mL, and most reactivations occurring between 2 and 6 weeks after CAR-T [30]. While only seven (10%) patients met the institutional threshold for pre-emptive therapy, five (7%) were treated [30]. Glucocorticoid use for >3 days and the use of BCMA CAR-T were significantly associated with a higher risk of CMV reactivation. BCMA CAR-T recipients had a higher number of prior lines of therapy, including a higher rate of prior HCT, which likely increased their net state of immunosuppression [30]. Additionally, this study evaluated CMV-specific cell-mediated immunity (CMV-CMI) at baseline and weeks 2 and 4 post CAR-T and demonstrated lower CMV-CMI at week 2 compared to baseline, with recovery to baseline levels by week 4, a pattern more pronounced in patients who developed CMV reactivation [30].

Furthermore, the relationship between CMV and post-CAR-T mortality has been explored. In the large cohort of 230 patients receiving CD19 CAR-T aforementioned, CS-CMVi within 1 year post CAR-T was significantly associated with a higher risk of non-relapse mortality (OR 2.49), despite the overall low rate of CMV end organ disease (3%), highlighting the potential indirect effects of CMV in this patient population, similar to the allogeneic HCT recipients [24,31]. The association of any CMV reactivation with an increased risk of mortality was also demonstrated in two other studies [25,26]. These studies are limited by their retrospective nature, the heterogeneity of the CMV definitions used and of the CMV monitoring protocols (or lack thereof). In summary, CMV reactivations are common after CAR-T, but CS-CMVi and CMV end organ disease remain rare. Most CMV reactivations occur early on after CAR-T in the context of a dip in CMV-CMI and disproportionately affect patients with CRS/ICANS, particularly those receiving glucocorticoids, and possibly recipients of BCMA CAR-T compared to CD19 CAR-T. At this time, CMV monitoring could be considered in the first 2 to 6 weeks after CAR-T in these higher risk patients [13]. While CMV reactivations have been associated with a higher risk of mortality, including non-relapse mortality, in CAR-T recipients, a direct causal relationship is still not established, particularly given the low rates of CMV end organ disease. Adequately powered prospective studies with systematic CMV monitoring are necessary to definitively assess the impact of CMV on overall and non-relapse mortality after CAR-T. Whether pre-emptive or prophylactic antiviral therapy (with letermovir for example) in this patient population may alter outcomes such as CMV end organ disease or all-cause mortality, as it does in allogeneic HCT recipients [32,33], needs to be determined in future trials.

### 2.3. HHV-6

Several cases of encephalitis secondary to HHV-6 after CAR-T have been reported [26,34,35,36], as well as a case of fatal HHV-6 myelitis with ascending flaccid paralysis and neuromuscular respiratory failure [37]. The diagnosis of HHV-6 encephalitis after CAR-T is challenging since the clinical presentation may overlap with ICANS [36]. Studies on the prevalence and significance of HHV-6 reactivation and risk of encephalitis after CAR-T are sparse. In a retrospective cohort of 230 CD19 CAR-T recipients, we identified 13 (6%) patients with HHV-6 reactivation but only 1 patient with HHV-6 encephalitis, when testing for HHV-6 was at the discretion of the treating provider [38]. Similar results were reported in a prospective study of 84 CAR-T recipients (including CD19 and BCMA CAR-T) with baseline and weekly plasma HHV-6 PCR testing up to week 12 post CAR-T. The cumulative incidence of HHV-6 reactivation was 6%, with all reactivations occurring between 2 and 6 weeks post CAR-T, and with no cases of encephalitis [39]. While neither study systematically tested cerebrospinal fluid for HHV-6, the low rate of clinical encephalitis leading to testing is encouraging.

While HHV-6 encephalitis appears to be rare after CAR-T, other potential manifestations of HHV-6, such as pneumonitis and its indirect effects, remain to be determined [40]. Additionally, the clinical significance of HHV-6 DNAemia is complicated by inherited chromosomal integration, for which testing is not yet widely available, and may explain a proportion of HHV-6 detections [39]. Interestingly, CAR T cells may super express HHV-6 in patients in vivo, postulating that cellular therapy products could be the source of transmission of HHV-6 in some of these patients [41]. The extent of this phenomenon remains unknown, since primary HHV-6 infection is quite common and typically occurs during childhood [40,42]. At this time, routine monitoring for HHV-6 after CAR-T is not recommended, and testing should be guided by clinical suspicion for end organ disease, particularly in CAR-T recipients with central nervous system symptoms and no alternative diagnosis or with poor response to ICANS treatment [13,40].

## 3. Bispecific Antibodies

Data on herpesvirus reactivations after BsAb are scarce, due to the limited reporting of infections in the clinical trials for these agents [43,44]. Additionally, many BsAb were approved through accelerated pathways, based on phase 1/2 trials with heterogeneous patients, smaller sample sizes, and varying dosing schedules [45].

Multiple platforms of BsAb are available or in development, with the bispecific T-cell engager (BiTE) being the most widely recognized [46]. The most common T cell epitope in BsAb is CD3, while the tumor target epitope varies depending on the tumor [46]. As such, blinatumomab, the first approved BsAb for B-ALL, is an anti-CD19xCD3 BsAb [46]. An alternative tumor antigen for B-cell lymphoma is CD20 [46]. In multiple myeloma, some of the BsAb constructs target BCMA (teclistamab, erlanatamab), whereas others target non-BCMA epitopes on plasma cells such as GPRC5D (talquetamab) [47,48,49]. Similarly to CAR-T, BsAb therapy has been associated with CRS and neurotoxicity, although at lower rates and severity. These complications may require further immunosuppression with IL-6 blockade and glucocorticoids, in patients who have already received multiple lines of therapy prior to BsAb and can be profoundly immunosuppressed [46]. Additionally, CD19/20- and BCMA-targeting BsAb have been associated with hypogammaglobulinemia, as an on-target, off-tumor effect [46], with depletion in IgG1 and IgG3 subclasses associated with an increased risk of CMV [50,51]. Prolonged cytopenias have also been described after BsAb, as a result of their inadvertent activation of regulatory T cells [52]. Furthermore, persistent antigen exposure and continuous CD3 receptor signaling have been associated with T-cell exhaustion during BsAb therapy, which, combined with the dampening of cytotoxic T-cell function by activated regulatory T cells, can explain an increased risk of viral infections, including herpesvirus reactivations [53,54]. Treatment-free intervals can improve T-cell exhaustion related to continuous BsAb exposure [54].

On the other hand, the lack of standardized reporting of infections in clinical trials and real-world studies for BsAb remains a significant concern as it may confound or delay the appropriate diagnosis of infections and its impact on outcomes.

### 3.1. Blinatumomab

In a phase 3 trial comparing blinatumomab, an anti-CD19xCD3 BsAb approved for B-ALL [46], to chemotherapy in B-ALL, fifteen patients (6%) in the blinatumomab arm had oral herpes, compared to nine patients (8%) in the chemotherapy arm; one patient had grade ≥ 3 herpes zoster and one patient had grade ≥ 3 oral herpes, both in the blinatumomab arm [55]. As a result, HSV/VZV antiviral prophylaxis is recommended in patients on blinatumomab [56]. In a phase 2 single-arm trial of dasatinib with glucocorticoids followed by blinatumomab in 63 patients with B-ALL, seven patients had grade ≥ 2 CMV infections [57], while it is worth noting that dasatinib alone has been associated with CMV colitis [58]. Data are too limited to make a recommendation regarding CMV monitoring in these patients.

### 3.2. BsAb in Patients with Lymphoma

Three anti-CD20xCD3 BsAb are currently approved for relapsed/refractory B-cell lymphomas, epcoritamab, glofitamab and mosunetuzumab [45]. A meta-analysis of infections in early phase clinical trials and observational studies of BsAb for lymphoma included 2228 patients and reported nine HSV/VZV reactivations, two CMV reactivations (one of which was deemed fatal) and two EBV reactivations across all four BsAb products included [45]. A single-center retrospective study of 44 patients with relapsed/refractory non-Hodgkin’s lymphoma treated with mosunetuzumab reported three episodes of herpes zoster [59].

### 3.3. BsAb in Patients with Multiple Myeloma

Teclistamab, an anti-BCMAxCD3 BsAb, is the first approved BsAb for the treatment of multiple myeloma, based on the phase 1/2 MajesTEC-1 trial [47,60]. A detailed analysis of infections in this trial was published 3 years after the original trial publication and included 165 patients who received teclistamab, 93% of whom received HSV/VZV prophylaxis [61]. Four (2%) patients developed oral HSV infection, three (2%) patients developed herpes zoster infection and three (2%) patients had CMV infections [54]. Erlanatamab is another anti-BCMAxCD3 BsAb approved for multiple myeloma. A total of 224 patients enrolled in two phase 1 and 2 trials for erlanatamab, and CMV infection occurred in 14 (6%) patients, including 2 who had CMV pneumonia [48,62]. Most patients in the phase 2 trial received HSV/VZV prophylaxis (87%) [48].

Talquetamab is an anti-GPRC5DxCD3 BsAb also approved for multiple myeloma, with the notable difference of targeting GPRC5D, an orphan receptor which is expressed on malignant plasma cells, unlike BCMA, which is expressed on malignant and healthy plasma cells, and mature B lymphocytes [49]. In a phase 1 trial, no CMV reactivations were reported in the 232 participants; however, 1 patient had disseminated VZV infection and 1 patient had ophthalmic herpes [49]. A pooled analysis of 1185 patients with multiple myeloma treated with BsAb monotherapy showed lower rates of neutropenia and of grade 3/4 infections in patients receiving non-BCMA BsAb compared to patients receiving BCMA-targeted BsAb [63]. This same analysis reported CMV infection and/or reactivation in 8% of the overall pooled cohort [63].

Few retrospective studies have offered additional perspectives to the early use of BsAbs in multiple myeloma (Table 2). In the majority of the studies, patients received HSV/VZV prophylaxis and, subsequently, HSV or VZV breakthrough infections were rare [64,65,66,67]. CMV reactivation at any level seemed more common, at an average of 10% (ranging from 3% [68] to 22% [69]). On the other hand, one study reported a prevalence of 11% for CS-CMVi, including two patients with CMV esophagitis and two patients with CMV PCR > 1000 IU/mL requiring pre-emptive antiviral therapy [69].

While the data on herpesvirus reactivations and associated risk factors after BsAbs for multiple myeloma remain largely descriptive and scarce, there is a consensus recommendation among multiple expert groups in favor of HSV/VZV prophylaxis during BsAb therapy and likely until immune reconstitution [70,71,72]. Even though the rates of CMV reactivation seem significant with reported cases of end organ disease, additional data are needed to determine the subset of patients at high-risk for CMV infections and who would benefit from targeted monitoring or prophylactic strategies. Finally, the burden and relevance of HHV-6 DNAemia after BsAb remain to be determined.

## 4. Future Directions

While antiviral prophylaxis for HSV/VZV in patients receiving CAR-T or BsAb became standard of care, a better understanding of immune recovery and vaccine response after either line of therapy is important to determine the appropriate indications and timing of recombinant zoster immunization and duration of antiviral prophylaxis in this patient population. Prospective studies to identify the subset of BsAb recipients at highest risk for CMV to develop appropriate CMV monitoring or preventive strategies in patients receiving CAR-T or BsAb and to evaluate the potential role of primary prophylaxis in the subset of high-risk patients are crucial. Finally, a better understanding of the role of HHV6 remains necessary in this patient population.

## Figures and Tables

**Table 1:** Studies of CMV reactivation after CAR-T.

CAR-T Product	Number of Patients	CMV Surveillance Protocol	Duration of Follow-Up	% with Any CMV Reactivation	% with CS-CMVi	% with CMV Disease	Risk Factors for CMV	Reference
CD19, BCMA	2256	None	Median 420 days	7.5%	N/A	N/A	Not reported	[11]
CD19	230	Weekly if neutropenia or grade 3/4 CRS/ICANS	365 days	22%	10%	3%	Asian/Middle Eastern Treatment for CRS/ICANS Meeting criteria for surveillance	[24]
CD19, BCMA	95	None	Median 352 days	33%	11%	0	2+ immunosuppressants	[25]
CD19	65	None	365 days	22%	15%	1.5%	Not reported	[26]
CD19	105	Weekly × 4 weeks	Minimum 30 days	44%	3%	0	Dexamethasone	[27]
CD19	51	Days 0, 7, 14, 21, 30, 60 and 90	90 days	56%	6%	0	Axicabtagene cileucel	[28]
CD19	60	Once at 14–21 days	30 days	17%	10%	0	Not reported	[29]
CD19, CD20, BCMA	72	Weekly × 12 weeks	12 weeks	27%	7%	0	Glucocorticoids > 3 days BCMA CAR-T	[30]

Abbreviations—CMV, cytomegalovirus; CAR-T, chimeric antigen receptor T-cell therapy; CS-CMVi, clinically significant CMV infection; N/A, not available; CRS, cytokine release syndrome; ICANS, immune effector cell-associated neurotoxicity syndrome.

**Table 2:** Studies of herpesvirus reactivations after BsAb.

BsAb Product	Number of Patients	% with Any CMV Reactivation	% with HSV/VZV	Reference
Any	39	18%	2%	[64]
Any	90	3% (No CMV disease)	-	[68]
BCMA	37	22% (11% CS-CMVi, 6% CMV disease)	-	[69]
BCMA	188	9%	0.5%	[65]
BCMA, GPRC5D	229	3.5%	1%	[66]
BCMA	55	4%	5%	[67]

Abbreviations—BsAb, bispecific antibody; CMV, cytomegalovirus; CS-CMVi, clinically significant CMV infection; HSV, herpes simplex virus; VZV, varicella zoster virus.
